# The distinction between state and trait anxiety levels in patients with BPPV in comparison with healthy controls

**DOI:** 10.3389/fpsyg.2022.1055467

**Published:** 2022-12-01

**Authors:** Liran Kalderon, Moshe Chaimoff, Michal Katz-Leurer

**Affiliations:** ^1^Sackler Faculty of Medicine, School of Health Professions, Department of Physical Therapy, Tel-Aviv University, Tel-Aviv, Israel; ^2^Clalit Health Services, Horowitz Physical Therapy Clinic, Tel-Aviv, Israel; ^3^Clalit Health Services, Migdal Hameah Health Center, Department of E.N.T, Tel-Aviv, Israel

**Keywords:** benign paroxysmal positional vertigo, anxiety, state–trait anxiety inventory, heart-rate, heart-rate variability

## Abstract

**Introduction:**

An association exists between vestibular dysfunction and anxiety, yet a distinction between state and trait anxiety in patients with Benign Paroxysmal Positional Vertigo (BPPV) in comparison with healthy subjects has not been well established. The aim of this study is to assess both state and trait anxiety levels in patients with BPPV in comparison with healthy controls, and their relations with cardiac autonomic parameters.

**Materials and methods:**

A case–control study, that included patients with BPPV (N = 18) referred to vestibular physiotherapy and gender and age matched healthy controls (N = 18). All participants completed the State–Trait Anxiety Inventory (STAI) questionnaire, while heart-rate (HR) and heart-rate variability (HRV) measures were recorded.

**Results:**

Patients with BPPV (age range 32–66 years; 12 women and 6 men) showed a higher state anxiety level (10 points median difference, *p* = 0.001) in comparison with healthy controls (age range 34–66 years; 12 women and 6 men), yet no differences were found in trait anxiety score or total STAI score. Only among patients with BPPV, a positive-moderate correlation was found between state anxiety and HR (*r* = 0.53, *p* < 0.05), and a negative moderate-strong correlation was found between state anxiety and HRV (*r* = −0.67, *p* < 0.01).

**Discussion:**

Patients with BPPV do not differ from healthy subjects in terms of predisposition to feel anxious, and only their temporary state anxiety levels are higher in comparison with healthy controls. We recommend researchers and clinicians who assess anxiety levels in patients with BPPV to distinguish between anxiety as a temporary state (state anxiety) and anxiety as a personality trait (trait anxiety).

## Introduction

Benign Paroxysmal Positional Vertigo (BPPV) is a one of the most common vestibular disorders, causing a burden on healthcare (Hanley et al., 2001; Parnes et al., 2003; von Brevern et al., 2007; Neuhauser and Lempert, 2009; Kollén et al., 2012; Kerrigan et al., 2013; Bhattacharyya et al., 2017). Patients who suffer from BPPV experience a false sensation of spinning (vertigo) provoked by head movements or positional changes, and commonly experience other symptoms, such as unsteadiness, nausea, vomiting, stress and anxiety (Jacob and Furman, 2001; von Brevern et al., 2015; Bhattacharyya et al., 2017).

An association between vestibular dysfunction and anxiety has been observed, and was suggested to co-occur through psychosomatic and somatopsychic mechanisms and overlapping neural circuits (Jacob and Furman, 2001).

It is acceptable to distinguish between anxiety as a temporary state, emphasizing an anxious reaction to a stressful stimulus, and anxiety as a personality trait, emphasizing a person’s predisposition to feel anxious in various situations (Julian, 2011). It has also been suggested that the two different types of anxiety are mapped differently in the human brain. Some consider trait anxiety to be related with structural grey matter volume changes, such as in the limbic regions, as well as with functional brain activity. Less is known about state anxiety and its relation to structural brain changes, but some evidence suggests it is associated with functional patterns of brain activity (Saviola et al., 2020).

The distinction between the two different types of anxiety levels in patients with BPPV in comparison with healthy subjects has not been well established. One study that did evaluate anxiety levels as a temporary state and anxiety as a personality trait among patients with BPPV, showed that both types of anxiety levels were decreased following vestibular physiotherapy treatment. However, anxiety levels were not compared to healthy controls (Gurpinar et al., 2020). It is therefore reasonable to question whether patients with BPPV have a predisposition to feel anxious, or is it only the stressful situation in which they experience during an onset of vertigo.

Anxiety levels are commonly evaluated by subjective measurements, such as questionnaires (Julian, 2011). A meta-analysis has shown that anxiety disorders are associated with reduced parasympathetic activity (Chalmers et al., 2014). Moreover, it is well known that a temporary emotional arousal is related to an increase in sympathetic activity (Spielberger, 1972; Macefield et al., 2013; Candia-Rivera et al., 2022). Heart-rate (HR) and heart-rate variability (HRV) measures are objective methods to quantify cardiac autonomic nervous system regulation. It has been shown that there is a negative correlation between anxiety levels and HRV measures in various populations (Chalmers et al., 2014).

The primary aim of this study is to assess both the state and the trait anxiety levels in patients with BPPV in comparison with healthy controls. A secondary aim is to assess the correlation between the state and the trait anxiety levels and HR and HRV measures in patients with BPPV in comparison with healthy controls. We hypothesized that patients with BPPV will present higher state anxiety levels than those observed in healthy controls, and similar trait anxiety levels to those observed in healthy controls. Additionally, we hypothesized that increased HR and reduced HRV will be only correlated with levels of state anxiety among patients with BPPV.

## Materials and methods

### Study design

This is a case–control study.

### Participants

The study took place in a public outpatient physiotherapy clinic (Clalit Health Services) in Tel-Aviv, Israel, between the years 2019–2020. A brief recruitment ad was published in the physiotherapy clinic where the study was conducted, and 36 participants were recruited for this study-18 Patients with BPPV and 18 age and gender-matched healthy controls.

#### Inclusion criteria

The study group included patients with BPPV between ages 30–70. The diagnosis of BPPV was confirmed by a subjective complaint of a spinning sensation (vertigo) elicited by head motion relative to gravity (such as when changing position in bed or bending over), and an objective positive Dix-Hallpike or Supine Roll test (presence of positional nystagmus; Bhattacharyya et al., 2017). The control group included healthy participants with no complaints of dizziness matched by age and gender. Participants in both groups were included after informed consent was obtained.

#### Exclusion criteria

(1) Diagnosis of any anxiety disorder; (2) Vestibular disorders that are not BPPV; (3) Head trauma or whiplash within the last year; (4) Complaints of any type of pain during the day of the visit; (5) Central neurological conditions, Autonomic nervous system disorders, Cardiovascular conditions or Beta-Blockers intake; (6) Medical conditions that are contraindicated for vestibular physiotherapy treatment; (7) Healthcare practitioners who are qualified to assess and treat vestibular disorders.

### Tests and measurements

#### Medical history, demographics and characteristics of symptoms

Were obtained and confirmed from subjects’ medical files and from patients’ self-report. As part of assessing patients’ characteristics-severity of symptoms and their impact on daily life, the *Hebrew version of the Dizziness Handicap Inventory (DHI)* questionnaire was used (Kaplan et al., 2010; Mutlu and Serbetcioglu, 2013). The DHI is validated with Short Form-36 (SF-36; Pearson’s correlation coefficient = 0.53–0.72; Fielder et al., 1996). Total score was classified as mild (0–30), moderate (31–60) or severe (61–100; Whitney et al., 2004).

#### State–trait anxiety inventory (STAI) for adults (form Y)

A questionnaire with two parts aimed to separately assess the level of state anxiety and trait anxiety. Each part includes 20 items with four-point ranks, and a higher total score of each part indicates a higher anxiety level. The questionnaire was validated with the Taylor Manifest Anxiety Scale and Cattell and Scheier’s Anxiety Scale Questionnaire (correlation coefficients are 0.73 and 0.85 respectively; Julian, 2011).

#### Dix-Hallpike test and supine roll test

The clinical tests that confirm the diagnosis of BPPV and known to elicit sensation of vertigo (Bhattacharyya et al., 2017). Positive predictive value of the Dix-Hallpike Test is 83.3%, the negative predictive value is 52.2%, and inter-rater reliability is high (Kappa = 0.92 [0.87–0.98], 95% confidence intervals; Hanley and Dowd, 2002; Burston et al., 2012).

#### Average HR and HRV-standard deviation of the normal-to-normal intervals (SDNN)

Monitored with a POLAR RS800CX (Polar Electro OY, Kempele, Finland) hand watch and a chest strap. Sampling rate was 1,000 Hz, and analysis of the recorded data was done with the Polar ProTrainer 5 software. The POLAR RS800CX is considered reliable, and has been validated with Electrocardiogram (Sztajzel, 2004; Nunan et al., 2009).

### Procedure

All patients with BPPV that were referred to vestibular physiotherapy evaluation and treatment, as recommended by their doctors, were offered to participate in the study. Among 20 patients who were referred with a diagnosis of BPPV, two patients refused to participate in the study due to lack of time. Healthy subjects who volunteered to participate in the study were assigned to the control group, matched by age and gender. The Ethics Review Boards of Clalit Health Services and Tel-Aviv University approved this study (NCT number 03867019), and written informed consent was obtained from all participants. The study was performed by a certified vestibular physiotherapist during a single session.

After wearing the Polar watch and chest strap, participants’ demographics and medical history were taken. Afterwards, diagnostical screening tests (the Dix-Hallpike and the Supine Roll tests) were performed. The examiner carefully observed the participants’ eyes to detect nystagmus (Bhattacharyya et al., 2017). If no evidence of nystagmus was found, participants with suspected BPPV were not included in the study group. Likewise, in the control group, none of the participants had positional nystagmus or reported dizziness during the screening tests. After the screening tests, only patients with BPPV were treated with the appropriate Canalith Repositioning Procedure (e.g., Epley maneuver), based on the Clinical Practice Guidelines for assessment and treatment of BPPV (Bhattacharyya et al., 2017). At the end of the meeting, all participants were asked to complete the DHI questionnaire and then the STAI questionnaire. When finished, heart rate recording was stopped.

### Statistical analysis

The *χ^2^* test was used to assess differences between groups for nominal variables. The Mann–Whitney test was used for ordinal variables and ratio variables with non-normal distribution. The Student’s T-Test was used for independent samples of ratio variables that were normally distributed, which was assessed by the Kolmogorov–Smirnov test and the Levene test for homogeneity of variances. When differences between groups were significant, the effect size was presented as well (Tomczak and Tomczak, 2014). Associations between anxiety levels and HR, and between anxiety levels and HRV were assessed with the Spearman’s correlation coefficient. To evaluate possible confounding effects, non-parametric partial correlation was examined.

Statistical analysis was performed with IBM SPSS Statistics (version 24), and statistical significance was assumed if *p* < 0.05, based on a two-tailed test.

## Results

Table 1 summarizes the demographics and DHI questionnaire scores.

**Table 1:** Demographics and DHI questionnaire scores of patients with BPPV in comparison with healthy controls.

		Patients with BPPV (N = 18)	Healthy controls (N = 18)	*p*-value
	Age (years)^1^	51.8 ± 10.9	51.3 ± 10.0	0.89
	Gender (women)^2^	12 (66.7)	12 (66.7)	1.00
	Total DHI score (/100)^3^	49 [4–90]	0 [0–0]	<0.01
	Physical DHI sub-score (/28)^3^	20 [2–26]	0 [0–0]	<0.01
	Functional DHI sub-score (/36)^3^	21 [0–36]	0 [0–0]	<0.01
	Emotional DHI sub-score (/36)^3^	12 [2–28]	0 [0–0]	<0.01

No significant differences were found in demographic characteristics between groups, yet DHI scores were significantly higher among patients with BPPV.

DHI-Dizziness Handicap Inventory_;_ BPPV-Benign paroxysmal positional vertigo; ^1^Ratio scale variables are shown as mean ± SD, *p*-values calculated with Student’s T-test, ^2^Nominal scale variables are shown as number (%), *p*-values calculated with χ^2^ test, ^3^Ordinal scale variables are shown as median [minimum - maximum], *p*-values calculated with Mann–Whitney test.

All patients with BPPV were diagnosed with a unilateral Canalithiasis form of BPPV-15 Patients (83.3%) were diagnosed with a posterior Semicircular Canal BPPV, two patients (11.1%) had a horizontal Semicircular Canal BPPV, and one patient (5.6%) had a mixed unilateral posterior and horizontal Semicircular Canal BPPV. Seven patients (38.9%) with BPPV reported a feeling of nausea on the day of assessment, and only two patients (11.1%) took medications to relieve symptoms of vertigo on the day of assessment (Cinnarizine or Betahistine). All patients who recently used medications to relieve symptoms of vertigo (27.8%) showed a median DHI score (functional subscale) of 28 [20–34], whereas other patients with BPPV showed median score of 14 [0–36] (*p* = 0.019). However, no differences were found in other variables.

Table 2 summarizes the anxiety levels, HR and HRV measures in patients with BPPV in comparison with healthy controls.

**Table 2:** Anxiety levels, heart-rate and heart-rate variability measures in patients with BPPV in comparison with healthy controls.

	Patients with BPPV (N = 18)	Healthy controls (N = 18)	*p*-value
**Anxiety levels**			
Total STAI score (/160)	68 [45–102]	62 [44–94]	0.09
State anxiety sub-score (/80)^1^	34 [20–67]	24 [20–43]	<0.01
Trait anxiety sub-score (/80)^1^	33 [23–53]	39 [24–54]	0.52
**Heart-rate and heart-rate variability**			
Average heart-rate (beats per minute)^2^	71.6 ± 12.2	69.5 ± 8.5	0.55
SDNN (milliseconds)^2^	53.0 ± 29.6	58.4 ± 21.4	0.53

BPPV-Benign paroxysmal positional vertigo; STAI-State Trait Anxiety Inventory; SDNN-Standard deviation of the normal-to-normal intervals; ^1^Ordinal scale variables are shown as median [minimum - maximum], *p*-values calculated with Mann–Whitney test; ^2^Ratio scale variables are shown as mean ± SD, *p*-values calculated with Student’s *T*-test.

A significant difference in the median state anxiety score between groups was found (*p* = 0.001). Patients with BPPV showed a higher state anxiety level (10 points median score difference) than healthy controls, with an effect size of 0.52. No significant differences between groups were found in median trait anxiety score, median total STAI score, average HR and average SDNN values.

Table 3 summarizes the correlations between anxiety levels and HR and HRV measures in patients with BPPV and in healthy controls.

**Table 3:** Correlations between anxiety levels and heart-rate/heart-rate variability measures in patients with BPPV and in healthy controls.

Anxiety levels		Patients with BPPV (N = 18)		Healthy controls (N = 18)	
		Correlation with HR	Correlation with HRV	Correlation with HR	Correlation with HRV
State anxiety	r _s_	0.53	−0.67	0.07	0.06
	*p*-value	*p* = 0.020	*p* = 0.002	*p* = 0.781	*p* = 0.826
Trait anxiety	r _s_	0.12	−0.02	−0.24	0.23
	*p*-value	*p* = 0.623	*p* = 0.929	*p* = 0.340	*p* = 0.340

BPPV-Benign paroxysmal positional vertigo; HR-Heart-rate, HRV-Heart-rate variability; r _s_-Spearman’s Correlation Coefficient.

Only among patients with BPPV, a positive-moderate correlation was found (r_s_ = 0.53, *p* = 0.02) between state anxiety levels and HR values, and a negative moderate-strong correlation was found (r_s_ = −0.67, *p* = 0.002) between state anxiety levels and HRV (SDNN values).

## Discussion

The main finding of this study is that patients with BPPV do not have higher trait anxiety levels than controls, meaning that patients with BPPV do not differ from healthy subjects in terms of predisposition to feel anxious. It was found that only state anxiety levels in patients with BPPV were higher in comparison with healthy controls.

These outcomes are consistent with findings in the literature suggesting presence of anxiety in conjunction with vestibular disorders (Jacob and Furman, 2001). It is also consistent with findings from another study that evaluated anxiety levels in patients with BPPV, where patients were admitted to an otorhinolaryngology outpatient clinic, using the Beck Anxiety Inventory (Kahraman et al., 2017). The researches evaluated anxiety levels at the initial visit and at two follow-up visits (7 days and 14 days after the first visit). The results showed that in comparison with healthy controls, patients with BPPV demonstrated higher anxiety levels at all three evaluations, especially at the initial visit. Furthermore, anxiety level was decreased at the follow-up visits after patients were treated with Canalith Repositioning Treatment. This finding is consistent with another study that evaluated anxiety levels after such treatment (Gunes and Yuzbasioglu, 2019). It can be therefore concluded that the results from our study not only strengthen the link between anxiety and BPPV, but also clarify that the anxiety experienced by the patients is a temporary state of anxiety, and not their personality trait. Consequently, clinicians and patients with BPPV may better understand the prognosis when assessing psychological factors (Wei et al., 2018).

This study also showed that only among patients with BPPV a positive-moderate correlation was found between state anxiety and HR, and a negative moderate-strong correlation between state anxiety and HRV. In other words, increased state anxiety levels are associated with increased sympathetic activity, as demonstrated by increased HR and reduced HRV. This finding is consistent with the expected physiological sympathetic response to a temporary emotional arousal (Spielberger, 1972; Macefield et al., 2013; Candia-Rivera et al., 2022). A meta-analysis that evaluated the association between anxiety and HRV in the general population, has also found a negative correlation between these two variables (Chalmers et al., 2014). In the current study no differences were found between groups in trait anxiety, and no correlations were found between trait anxiety and HR and HRV in both groups. However, a moderately strong association was noted between HRV and anxiety only among patients with BPPV merely in the state anxiety factor. It seems that the results of this study strengthen the assumption that patients with BPPV only experience a temporary state of anxiety.

### Study limitations

The main limitation of the current study is that no data of anxiety levels, HR and HRV measures was collected on follow-ups, when major resolution of vertigo symptoms is expected to occur a few days after vestibular physiotherapy treatment (Bhattacharyya et al., 2017). Documentation of such data on follow-ups could have validated the findings of this study, and future studies should target this point. Another limitation of this study is the relatively small sample size when assessing differences in correlations between groups. Based on the correlations observed in the current study between anxiety levels and HR and HRV measures in patients with BPPV and healthy controls, the sample size needed to present significant between-group differences varied between 24 participants (for HR) to 36 participants (for HRV) in each group (Bonett and Wright, 2000).

## Conclusion

In summary, patients with BPPV do not seem to differ from healthy subjects in terms of predisposition to feel anxious. The current study clarifies that only state anxiety levels seem to be higher in patients with BPPV during an onset of vertigo as compared to healthy controls. We recommend researchers and clinicians who assess anxiety levels in patients with BPPV, or describe the link between anxiety and vestibular dysfunction, to distinguish between anxiety as a temporary state (state anxiety) and anxiety as a personality trait (trait anxiety).

## Data availability statement

The raw data supporting the conclusions of this article will be made available by the authors, without undue reservation.

## Ethics statement

The studies involving human participants were reviewed and approved by Clalit Health Services and Tel-Aviv University Ethics committees. The patients/participants provided their written informed consent to participate in this study.

## Author contributions

All authors contributed to the study conception and design. This scientific work was performed by LK in partial fulfillment of the M.Sc.PT thesis requirements of the Sackler Faculty of Medicine, Tel Aviv University. This work was done in Horowitz Physical Therapy Clinic, Clalit Health Services, Tel-Aviv, Israel. All authors contributed to the article and approved the submitted version.

## Conflict of interest

The authors declare that the research was conducted in the absence of any commercial or financial relationships that could be construed as a potential conflict of interest.

## Publisher’s note

All claims expressed in this article are solely those of the authors and do not necessarily represent those of their affiliated organizations, or those of the publisher, the editors and the reviewers. Any product that may be evaluated in this article, or claim that may be made by its manufacturer, is not guaranteed or endorsed by the publisher.

## Abbreviations

BPPV: Benign paroxysmal positional vertigo
DHI: Dizziness handicap inventory
HR: Heart-rate
HRV: Heart-rate variability
SDNN: Standard deviation of the normal-to-normal intervals
STAI: State–trait anxiety inventory
